# The Last of Us and the Question of a Fungal Pandemic in Real Life

**DOI:** 10.3201/eid3003.230684

**Published:** 2024-03

**Authors:** Georgios Pappas, Georgia Vrioni

**Affiliations:** Institute of Continuing Medical Education of Ioannina, Ioannina, Greece (G. Pappas);; National Kapodistrian University of Athens, Athens, Greece (G. Vrioni)

**Keywords:** pandemic, fungi, *Cordyceps*, The Last of Us, television series, public perception

## Abstract

The television series The Last of Us imagines a postapocalyptic world ravaged by a fungal pandemic caused by a *Cordyceps* species. We evaluate whether a fungal pandemic is possible (and reasons behind its current improbability). We further discuss the series’ effect on public perception of fungi, fungal infections, and pandemic response.

The recent release of The Last of Us, a television drama series created for HBO consisting of 9 episodes in its first season (and renewed for a second season), has shed light on the global significance of fungal infections and spurred discussions on their potential to cause a pandemic. The series has met with wide acclaim, even prompting the Centers for Disease Control and Prevention to officially clarify the plausibility of the show’s premise in a tweet. Created by Craig Mazin and Neil Druckmann, The Last of Us is based on a successful video game developed in 2013 by the company Naughty Dog. Both the game and the television series take place in a postpandemic world, in which most humans have been either transformed into zombies by a human-adapted, mind-controlling fungal species of *Cordyceps* or killed by zombies, rogue humans, or the totalitarian state. Twenty years after the outbreak, a young girl who is immune to infection crosses the United States, accompanied by her protector, to reach scientists hoping to create a cure or a vaccine by studying her.

## Is Such a Scenario, of a Fungal Pandemic, Plausible?

Up to 5.1 million fungal species are estimated to exist in nature (*1*). About 148,000 types have been characterized, a few hundred of which are pathogenic for humans (*2*). A recent fungal priority pathogens list developed by the World Health Organization attributes 1.6 million annual deaths to fungal infections (*3*); considerable illness can also be attributed to fungal infections. In recent years, a rising percentage of emerging infectious diseases has been fungal in nature, including multidrug-resistant species with considerable mortality such as *Candida auris* (*4*) and rapidly disseminating ones such as *Trichophyton indotineae* (*5*). In a planetary health approach, the significance of fungal infections is even broader. Eighty percent of plant diseases are attributed to fungi, including pathogens that bring about substantial species or crop destruction worldwide. *Cryphonectria parasitica* eliminated almost 4 billion sweet chestnut trees in the eastern United States after its geographic introduction (*6*), *Magnaporthe oryzae* has destroyed rice crops (*7*), and *Puccinia graminis* has emerged as a major risk for grains (*8*). Panzootics can be caused by fungi, even threatening to evolve into extinction-level events; a recent example is the emergence of chytrid fungi that have menaced numerous amphibian species (*9*).

In humans, the importance of fungal infections has been increasing because of the increase in susceptible populations, in particular immunocompromised persons of varying immunologic deficits, ranging from transplant patients to persons with diabetes mellitus (which is known to predispose persons to severe mucormycosis) (*10*). Progress in antifungal therapeutic interventions has been slow, partly because of the fungi eukaryotic nature, which can lead to substantial adverse events. At present, only 4 classes of antifungals are available (azoles, polyenes, pyrimidines, and echinocandins), although research toward new antifungal development is promising (*11*). Certain species express a multidrug-resistant profile, though, including *C. auris* and *T. indotineae*.

Selective pressures might account for emergence of novel fungal pathogens, as in the case of *C. auris*, the concurrent worldwide appearance of which might be a consequence of global warming, enabling fungal species to adapt to higher temperatures and subsequently to human body temperature, a major obstacle to the development of nonsuperficial fungal infections in humans (*2*,*12*). Human practices also induce fungal reemergence, as with the appearance of resistant *Aspergillus* species because of the extensive, uncontrolled use of fungicides in agriculture (*13*).

Fortunately, fungi are relatively slow mutators. The process of species-jumping and host adaptation, such as in the case of *Ophiocordyceps unilateralis* (the prototype for the pathogen in The Last of Us), which adapted from beetle-infecting species to ant fungal pathogen (*14*), is time consuming and would not be expected to occur over just a few years.

*Cordyceps* species are ubiquitous: >100 have been described, they are species-specific, and >35 of them perform “mind control” in their hosts. The *Cordyceps* name is derived both from Ancient Greek and Latin: κορδύλη means truncheon and *ceps* means head. *O. unilateralis*, upon infecting an ant, modifies the host’s behavior, leading the ant to move to a specific tree-branch height before it dies; the fungus then destroys the host body and sheds fungal spores (from an ideal height) for further fungal dissemination in the environment.

No vertebrate *Cordyceps* hosts exist, and an evolutionary path leading there would probably require tens of thousands of years. Other brain-modifying or brain-occupying pathogens do exist, however, such as rabies virus, perhaps the most typical. Human behavior can be modified by pathogens to enable their spread in simpler ways: common cold viruses induce coughing and sneezing, essentially enhancing their own transmission, and similarly, gastrointestinal pathogens change human bowel habits and enable them to spread through diarrhea (*15*). Further focusing on neural involvement, primary amoebic meningoencephalitis, caused by *Naegleria fowleri*, might be a more accurate example of a brain-eating pathogen. Bornavirus has in the past been considered a cause of psychiatric disorders (an outcome of brain modification), and the role of toxoplasmosis in the future development of schizophrenia has also been evaluated. Numerous other pathogens can manifest through chronic central nervous system involvement and neuropsychiatric symptomatology, including the fungi *Cryptococcus neoformans*.

The extraordinary success of The Last of Us has implications, because all depictions of epidemics and infection in film and television can affect public perceptions of infectious diseases and outbreaks (*16*,*17*). The video game itself was partly successful because it described a critical dystopia (*18*) but one that included utopian foci that signify hope and resistance (in contrast to classical dystopias) and act as a pathway to catharsis, an escape from the doom, for the player and, subsequently, the viewer. In addition, the game was scripted with valid scientific details and an openness to moral issues (*19*): the enemies were not only the infected persons who had become zombies. The Federal Disaster Response Agency was also an enemy, because it represented a totalitarian force that had little to do with public health and protection (admittedly, this is a television show betting on horror and serves as a worst-case scenario and pessimistic study in social psychology). But surviving humans also, at times, became enemies out of desperation or vile evolution (e.g., the Raiders, survivor gangs attacking other uninfected humans for food and supplies). Even the Fireflies, the citizen group fighting the totalitarian state, could be considered an enemy because their mission includes killing the immune child to use her brain to prepare a vaccine. As Erik English recently stated (*20*), sacrificing a child for the greater societal good represents a broken social contract.

The series is ambitious in its scientific statements to the extent that they align with a compelling narrative. Thus, whereas major scientific issues such as global warming, pandemics, and accelerated mutation and adaptation of pathogens are discussed (things that many viewers with a casual understanding of science will recognize as potential threats even if they do not understand the pathology of fungi), certain details might succumb to the needs of the narrative. The series begins with a televised expert panel discussion in the late 1960s; an expert explains that although humanity has been at constant war with epidemic- and pandemic-causing viruses and bacteria, that war is, eventually, always won, despite casualties and lost battles. However, the same would not be certain if a fungal enemy emerged because of climate change, the expert warns.

Fast forward to the opening of the second episode, which narrates the initial outbreak in Indonesia, describing how the epidemic started in a grain/flour factory, initially infecting persons in contact with infected products but then rapidly disseminating through person-to-person transmission worldwide. This point is where the need of the show runners to impress the viewer diverts from scientific reasoning: apart from the improbably fast dissemination of the nonairborne pathogen worldwide, the series presents an expert Indonesian mycologist who states, when asked what should be done about the outbreak, “Bomb Jakarta,” an awe-inducing statement. Bombing was implied as a means of outbreak containment in the 1995 film Outbreak, considered to be one of the most accurate on-screen depictions of an outbreak (*16*), but in that scenario, at least, the army proposed it, whereas here it is a scientist’s proposal. One could argue that if Jakarta were bombed in this hypothetical scenario, humanity could have been spared from the apocalypse. However, this statement immediately renders the scientific community useless, possibly indirectly weakening the public’s trust in science itself (or reflecting public worries about the ability of science to respond adequately). Similarly, the fact that the human response to the pandemic eventually led to a totalitarian state (complete with quarantine zones and death penalties) might reflect the audience’s actual fears, particularly in the context of an actual pandemic, in which necessary initial lifesaving measures (e.g., lockdowns) have been vilified by merchants of disinformation. (One could counter-argue that certain approaches to viral containment in China were, or have been presented in the world media as, dystopic). The choice of Jakarta as the origin of the pandemic might feed inaccurate stereotypes that link emerging infectious diseases specifically with the developing world, but southeast Asia has no relevant outbreak history of emerging fungal infections and would not be considered a fungal hot spot. Jakarta could be considered a megacity, however, and as such could contain areas with hygienic challenges that could favor early infection dissemination.

The Last of Us is not the first work of art depicting a postapocalyptic world caused by a *Cordyceps* species adapted to humans. The 2016 film The Girl With All The Gifts, based on the Mike Carey book of the same title, imagines a world where the pathogen achieves equilibrium with its hosts, resulting in a society that breeds intelligent zombie children (“They had to live with the pathogen, endemicity was unavoidable” echoes the excuses used for our actual pandemic response fatigue). The initial depiction of a human-infecting *Cordyceps* outbreak, though, was in 2011, in the Fox television series Fringe, in an episode titled Alone in the World. In that episode, a variant of the fungus with the capacity for hyper-accelerated growth and nutrition absorption formed an extended neural network and was eventually contained with a specifically developed toxin (after initial partially successful ultraviolet light attempts).

Eventually, is a fungal pandemic a plausible scenario? Fungi are not included in the World Health Organization prioritization criteria for potential biologic weapon development and use, and other prioritization scores for biologic weapons (*21*) would yield a low score for fungi. There is no history of rogue research on fungal weaponization; in addition, a narrow spectrum of the population would be vulnerable to such a pathogen, and person-to-person transmission would be limited (we do inhale fungal spores, but we do not exhale them). On the other hand, a fungal pandemic would find humanity ill-prepared. Our diagnostic capacity for fungal pathogens remains extremely limited, no vaccines are available (although preliminary research has been conducted on a *Coccidioides* vaccine, and a *Candida* vaccine has been tested in a phase 2 clinical trial of vulvovaginal candidiasis) (*22*,*23*), and our therapeutic interventions are limited, costly, and have major side effects. Yet there would be space for preventive use of interventions: would rapid dissemination of antifungal medication be feasible in such a case? And how rapidly would antifungal resistance emerge?

In conclusion, The Last of Us might resonate with audiences because of our current experience with a pandemic unprecedented for the modern scientific world, in addition to the creators’ narrative abilities and the minor infusions of scientific accuracy. Does The Last of Us leave viewers with a perhaps dangerous and misconstrued perception about how the preparedness of the scientific and public health community to deal with pathogens and pandemics could lead society into an Orwellian dystopia? One could wish for future depictions of zombie apocalypses that are more optimistic regarding human behavior. An example of more positive messaging depicting such an event was the Center for Disease Control and Prevention Zombie Apocalypse preparedness exercise (now retired), which created a much more optimistic scenario while educating persons on how to be ready for an emergency. The Last of Us is not upon us, neither biologically nor psychologically; humankind’s response in reality might, we believe, be far kinder than what is portrayed here.
